# Identification of Estrogen Receptor-Related Receptor Gamma as a Direct Transcriptional Target of Angiogenin

**DOI:** 10.1371/journal.pone.0071487

**Published:** 2013-08-15

**Authors:** Jian Ang, Jinghao Sheng, Kairan Lai, Saisai Wei, Xiangwei Gao

**Affiliations:** 1 Institute of Environmental Medicine, Zhejiang University School of Medicine, Hangzhou, China; 2 Program in Molecular Cell Biology, Zhejiang University School of Medicine, Hangzhou, China; 3 Medical Class 2006, Zhejiang University School of Medicine, Hangzhou, China; 4 Department of Gastroenterology of the Second Affiliated Hospital, Zhejiang University School of Medicine, Hangzhou, China; Roswell Park Cancer Institute, United States of America

## Abstract

Nuclear translocation of angiogenin (ANG) is essential for the proliferation of its target cells. ANG promotes rRNA synthesis, while whether it regulates mRNA transcription remains unknown. Using the chromatin immunoprecipitation method, we have identified 12 ANG-binding sequences. One of these sequences lies in the estrogen receptor-related receptor gamma (ERRγ) gene which we designated as ANG-Binding Sequence within ERRγ (ABSE). ABSE exhibited ANG-dependent repressor activity in the luciferase reporter system. Down-regulation of ANG increased ERRγ expression, and active gene marker level at the ABSE region. The expression levels of ERRγ targets genes, p21^WAF/CIP^ and p27^KIP1^, and the occupation of ERRγ on their promoter regions were increased in ANG-deficient cells accordingly. Furthermore, knockdown of ERRγ promoted the proliferation rate in ANG-deficient breast cancer cells. Finally, immunohistochemistry staining showed negative correlation between ANG and ERRγ in breast cancer tissue. Altogether, our study provides evidence that nuclear ANG directly binds to the ABSE of ERRγ gene and inhibits ERRγ transcription to promote breast cancer cell proliferation.

## Introduction

Angiogenin (ANG) is a 14-kDa angiogenic protein originally isolated from the conditioned medium of HT-29 human colon adenocarcinoma cells based solely on its capacity to induce angiogenesis [1]. Its expression is up-regulated in various types of human cancer [2], indicating a close relationship between ANG and tumor development. ANG exerts angiogenic function by activating endothelial and smooth muscle cells and inducing a number of cellular activities, including cell migration, invasion, proliferation, and formation of tubular structures [3], [4]. Recently ANG has been reported to regulate the proliferation of cancer cells including HeLa cells and PC-3 cells directly [2], [5], indicating that ANG regulates the activities of both vascular cells and cancer cells during tumor development.

ANG undergoes nuclear translocation in its target cells, which is essential for angiogenesis and cancer cell proliferation. Either inhibition of nuclear translocation by neomysin [2], [6] or mutagenesis at the nuclear localization sequence [7] abolishes ANG-promoted cell proliferation. The nuclear ANG has been shown to bind to the rRNA gene (rDNA) and stimulate rRNA transcription catalyzed by polymerase I (Pol I) [8], [9], which determines ribosome biogenesis and protein synthesis [10]. During cell proliferation, ANG-stimulated rRNA synthesis must coordinate with the expressions of mRNAs catalyzed by Pol II. Lines of evidence also suggest a role of ANG in mRNA transcriptional regulation [2], [9]. However, the direct target genes of nuclear ANG remain elusive.

Chromatin immunoprecipitation (ChIP) is a potent method to identify novel target regulatory elements when combined with high throughput DNA analyzing methods such as cloning, arrays, and direct sequencing [11], [12]. The unbiased ChIP screening avoids the confusing side effects resulting from alterations of transcriptional pathways. Using the ChIP method combined with cloning and sequencing (ChIP-cloning), we identified several ANG-binding sequences, one of which lies within the *estrogen receptor-related receptor gamma (ERRγ)* gene.

ERRγ belongs to the nuclear receptor subfamily closely related to estrogen receptor. It can activate the transcription of target genes without estrogen binding. Recent studies have shown that the expression of ERRγ is down-regulated in breast cancer and prostate cancer [13], [14]. Over-expression of ERRγ inhibits cancer cell proliferation *in vitro* and xenograft tumor growth in mice [14], [15], implicating a repressive role of ERRγ in tumor development. The inhibitory effect of ERRγ on cancer cell proliferation was attributed to the induction of two cyclin-dependent kinase inhibitors p21^WAF/CIP^ and p27^KIP^ [14]. However, the regulation of ERRγ expression in cancer cells remains largely unknown. In this study, we further investigated the regulatory role of ANG in ERRγ expression in breast cancer cells.

## Materials and Methods

### Ethics Statement

The breast ductal carcinoma tissue microarray samples were obtained from US Biomax, Inc. (Rockville, MD, United States). All tissues were collected under the highest ethical standards with the donor being informed completely and with their consent. The company follows standard medical care and protects the donors’ privacy. All human tissues were collected under Health Insurance Portability and Accountability Act (HIPPA) approved protocols.

### Cell Culture

Human cervical carcinoma cells (HeLa), human breast cancer cells (MCF-7), and human prostate cancer cells (PC-3) were obtained from ATCC. HeLa and MCF-7 cells were maintained in DMEM (Invitrogen, Carlsbad, CA, United States) supplemented with 10% fetal bovine serum (Thermo Fisher Scientific, Waltham, MA, United States). PC-3 cells were maintained in RPMI1640 (Invitrogen) supplemented with 10% fetal bovine serum. Cells were maintained at 37°C in an atmosphere containing 5% CO_2_ and 100% humidity. Cell numbers were determined with a Coulter Counter for at least three biological repeats.

### ANG and Neomycin Treatment

HeLa or MCF-7 cells at 80% density were applied to exogenous recombinant human ANG or neomycin (Sigma-Alrich, MO, United States) treatment. Cells were washed with serum free medium and incubated with 1 ug/mL ANG at 37°C for 1 hour. For neomycin treatment, cells were pretreated with 100 μM neomycin for 30 min before exogenous ANG was added for another 1 hour. The treated cells were used for further studies.

### Immunofluorescence Staining

Cells grown on glass coverslips were rinsed with PBS and fixed in 4% formaldehyde in PBS for 15 minutes. After rinsing twice with PBS, the cells were permeabilized in 0.2% Triton X-100 in PBS. The cells were then incubated with anti-ANG antibodies (Santa Cruz Biotechnology, Santa Cruz, CA, United States) for 1 hour, stained with the tetramethyl rhodamine isothiocyanate (TRITC)-conjugated secondary antibody in PBS for 1 hour and mounted on microscope slides. Images were obtained by fluorescence microscopy.

### Chromatin Immunoprecipitation

ChIP assays were performed using the ChIP assay kit (Thermo Fisher Scientific) according to manufacturer’s protocol. Briefly, HeLa, MCF-7, or PC-3 cells were cross-linked with 1% formaldehyde for 10 minutes at 37°C. Cross-linking was stopped with 0.125 M glycine. The cells were collected and resuspended in lysis buffer (50 mM Tris-HCl at pH 8.1, 1% SDS, 10 mM EDTA, and protease inhibitors). After sonication to yield DNA fragments of 500 to 1000 base pairs, lysates were cleared by centrifugation, diluted tenfold with ChIP dilution buffer (16.7 mM Tris–HCl at pH 8.1, 0.01% SDS, 1.1% Triton X-100, 1.2 mM EDTA, 16.7 mM NaCl, and protease inhibitors) and precleared with salmon sperm DNA/protein G agarose at 4°C for 1 hour. For each immunoprecipitation assay, lysates were incubated with 5 μg of anti-ANG (Santa Cruz Biotechnology), anti-K4-dimethylated histone H3 (H3K4me2) (Thermo Fisher Scientific), anti- acetylated histone H4 (acetyl-H4) (Thermo Fisher Scientific), anti-RNA polymerase II (Abcam, Cambridge, MA, United States), anti-ERRγ (Santa Cruz Biotechnology), or control IgGs (Santa Cruz Biotechnology) overnight at 4°C with rotation. The immuno-complexes were then collected with protein G agarose slurry, eluted and de-crosslinked at 65°C. After RNase digestion and proteinase digestion, immunoprecipitated DNA was extracted with a QIAquick spin kit (Qiagen, Valencia, CA). The purified DNA was amplified by real-time PCR with the ABI7900 (Applied Biosystems, Foster City, CA, United States) and SYBR GREEN PCR Master Mix (Applied Biosystems). The primers used for amplifications were listed in Table S1.

Two sequential ChIPs were applied for the cloning assay as described previously [11]. Briefly, the eluted immune-complexes were re-diluted with ChIP dilution buffer and re-immunoprecipitated with anti-ANG. DNA fragments were purified, blunted and cloned into pUC19 vectors. Sequence from positive clones was obtained using the universal M13/pUC sequencing primer (5′-CGCCAGGGTTTTCCCAGTCACGAC-3′).

### Reporter Plasmids Construction

The ABSE sequence was cloned into SacI and HindIII sites of pGL3-enhancer vector (Promega, Madison, WI, United States) in the forward (ABSE+) and reverse (ABSE−) direction using the primers listed in Table S1.

### RNA Interference

To knock down the endogenous target protein, MCF-7 and PC-3 cells were transiently transfected with 10 nM of the chemically synthesized siRNAs using Lipofectamine2000 (Invitrogen) according to the manufacturer’s recommendations. Cells were harvested or treated for further experiments 48 h after transfection. siRNA sequences used in the present study are designed as follows: ANG siRNA1 (siANG-1), forward, 5′-AAGAAUGGAAACCCUCACAGA-3′, reverse, 5′-UCUCUGUGAGGGUUUCCAUUC-3′; ANG siRNA2 (siANG-2), forward, 5′-GCAUCAAGGCCAUCUGUGATT-3′, reverse, 5′-UCACAGAUGGCCUUGAUGCTG-3′; ERRγ siRNA (siERRγ), forward, 5′-GCCCAAGAGACUGUGUUUATT-3′, reverse, 5′-UAAACACAGUCUCUUGGGCTT-3′; negative control siRNA, forward, 5′-UUCUCCGAACGUGUCACGU-3′, reverse, 5′-ACGUGACACGUUCGGAGAA-3′. siRNAs were synthesized by GenePhama company (Shanghai, China).

### Luciferase Assay

MCF-7 cells were transfected with each firefly luciferase construct (control vector, ABSE+ and ABSE−) together with control siRNA or ANG siRNA1. *Renilla* reporter vector pRL-TK (Promega) was co-transfected as the internal control. Cells were lysed and luciferase activity was measured by the Dual-Luciferase assay system (Promega) 48 hours after transfection. The firefly luciferase activity was normalized to renilla luciferase.

### RNA Purification and Reverse Transcription qPCR

Total RNA was isolated from the MCF-7 and PC-3 cells with siRNA treatment by using Trizol reagent (Invitrogen) following the manufacturer’s protocol. RNA was reverse transcribed using random hexamers and the High Capacity cDNA Reverse Transcription Kit (Applied Biosystems).

Real-time quantitative PCR analysis was performed in 10 μL reactions using the ABI7900 (Applied Biosystems) and SYBR GREEN PCR Master Mix (Applied Biosystems). The primers used for *GAPDH* gene (GAPDH coding region) and *ERRγ* gene (ERRγ coding region) amplifications were listed in Table S1. Expression of *ERRγ* gene was normalized relative to *GAPDH* using the 2^−ΔΔCT^ method [16].

### Immunohistochemistry

Embedded breast ductal carcinoma tissues were deparaffinized with xylene, rehydrated in ethanol, and boiled in 10 mM citrate buffer (pH 6.0) for 30 min for antigen retrieval. Endogenous peroxidase was blocked by treatment with 3% H_2_O_2_. After blocking in goat serum for 30 min at room temperature, tissues were incubated with anti-ANG (Santa Cruz Biotechnology) or anti-ERRγ (Santa Cruz Biotechnology) at 4°C overnight. The slides were then visualized with Envision System (DAKO Corporation, Carpinteria, CA, USA) and counterstained with hematoxylin.

### Statistical Analysis

All data is expressed as the mean ± SD of three independent experiments. Statistical analysis was conducted with a double-sided Student’s *t* test or one-way ANOVA analysis of variance among groups. Values of *P*<0.05 were accepted as statistically significant.

## Results

### Identification of ANG-binding Sequences

**Figure 1:**
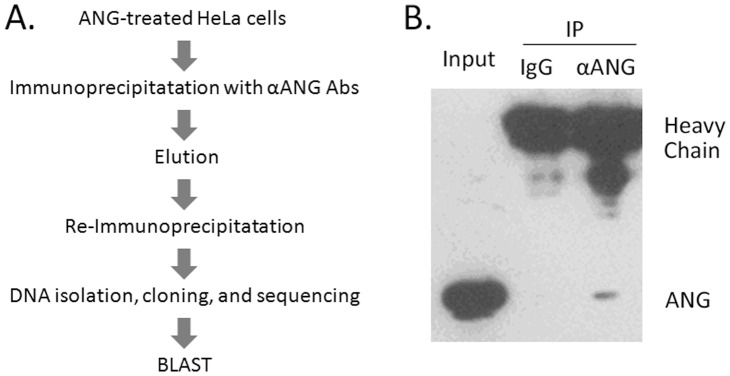
Identification of ANG-binding DNA fragments. (**A**) Schematic illustration of ChIP screen of ANG-binding DNA. (**B**) Sonicated chromatin samples from HeLa cells were immunoprecipitated overnight with ANG antibody or IgG and applied to Western blot analysis. Data showed specific enrichment of ANG in the antibody group.

Angiogenin (ANG) translocates to the nucleus of target cells, which is essential for angiogenesis and cancer cell proliferation. Nuclear ANG can localize in the nucleolus and promote the production of ribosomal RNA (rRNA). The previous reports suggest that ANG can also localize in the nucleoplasm [2], implying that ANG may regulate mRNA transcription. To identify novel, direct ANG target genes, we carried out an unbiased ChIP-cloning method (Fig. 1A). HeLa cells treated with ANG were fixed with formaldehyde, and chromatin DNA was sonicated to an average size of 600 base pairs (Fig. S1). ANG/DNA complexes were specifically immunoprecipitated by polyclonal anti-ANG antibodies as revealed by the presence of ANG in the antibody group but not in the IgG group (Fig. 1B). To enable more efficient cloning of the specific ANG-binding fragments, we performed two sequential chromatin immunoprecipitations using the same anti-ANG antibodies as described previously [11], and ANG-bound DNA was cloned into the plasmid vector pUC19 and sequenced. After mapping to the human genome using the BLAST algorithm, we totally identified 12 different ANG-interacting DNA fragments (Table 1). Among these fragments, three are located within introns, and nine are upstream or downstream of genes with a distance ranging from 927 bp to 273 kb, further supporting that ANG binds to regulatory elements and is involved in mRNA expression regulation. The sequences are shown in Table S2.

**Table 1:** Information of ANG-binding fragments identified by ChIP-cloning.

Clone	Length(bp)	Nearest gene	Accession number	Location to gene	Distance
1, 2	1160	Estrogen-related receptor gamma	NM_001438	First intron	
3	416	CASP2 and RIPK1 domain containing adaptor withdeath domain	NM_003805	5′	1615 bp
4	587	PDZ domain containing 6	NM_015693	Ninth intron	
5	891	peroxisome proliferator-activated receptor gamma,coactivator 1 alpha	NM_013261	3′	54 kb
6	212	CD226 antigen precursor	NM_006566	5′	927 bp
7	1026	Zinc finger protein 121	NM_001008727	5′	13 kb
8	128	PDE4D phosphodiesterase 4D, cAMP-specific	NM_006203	3′	107 kb
9	623	Protocadherin Fat 1 precursor	NM_005245	3′	126 kb
10	244	Kazrin A	NM_015209	Third intron	
11	620	Pleckstrin homology domain-containing family Fmember 2	NM_024613	5′	9 kb
12	957	Transcription factor EC isoform a	NM_012252	5′	273 kb
13	966	ADAM metallopeptidase with thrombospondintype 1 motif, 9	NM_182920	5′	201 kb

### Confirmation of ANG Binding to ABSE

**Figure 2:**
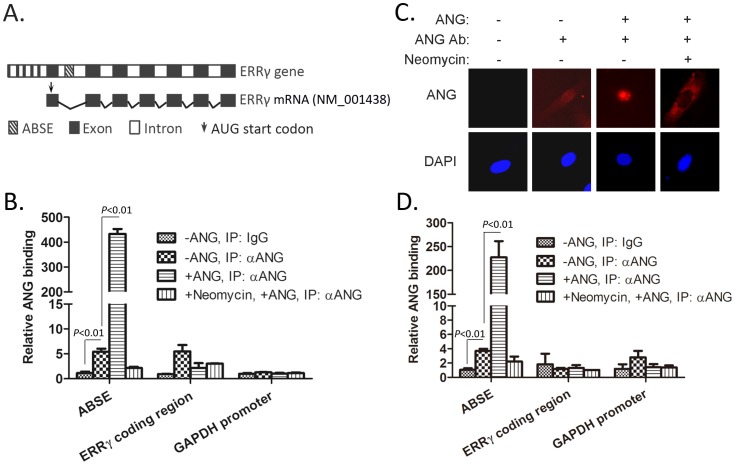
ANG interacts with ABSE fragment in HeLa and MCF-7 cells. (**A**) Schematic illustration of ABSE localization in *ERRγ* gene. (**B**) HeLa cells were pretreated with or without neomycin for 1 hour and incubated with 1 ug/mL of ANG. Cells were then applied to ChIP experiments with IgG or ANG antibody and analyzed by qPCR. Data shown represents mean±s.d. of three independent experiments. (**C**) MCF-7 cells were pretreated with or without neomycin for 1 hour and incubated with 1 ug/mL of ANG. Cells were then stained with ANG polyclonal antibodies. Nuclei were stained with DAPI (blue). (**D**) MCF-7 cells were treated with or without neomycin followed by incubation with 1 ug/mL of ANG. Cells were then applied to ChIP experiments. Data shown represents mean±s.d. of three independent experiments.

Notably, two of the fragments contained the same sequence (Table 1) mapping to a region within *ERRγ* gene. The location of this sequence within *ERRγ* gene was shown in Fig. 2A. We named this sequence *A*NG-*B*inding *S*equence within *E*RRγ gene (ABSE) and applied it to further analysis.

We first validated the association of ANG with the ABSE by using conventional ChIP-qPCR analyses in HeLa cells. Data showed that immunoprecipitation of endogenous ANG enriched chromatin fragments of ABSE, in comparison to IgG group. ANG treatment significantly increased the binding between ANG and ABSE. Neomycin treatment, which blocks ANG nuclear translocation [2], decreased the binding between ANG and ABSE comparable to basal level. No enrichments of ERRγ coding regions or control GAPDH promoter were observed in ANG immunoprecipitated samples (Fig. 2B), indicating that ANG specifically associated with ABSE in HeLa cells. The binding of ANG with ABSE sequence was further validated in MCF-7 cells. Immunofluorescence staining showed that ANG treatment significantly increased while neomycin treatment blocked the nucleolar and nucleoplasm localization of ANG in this cell line (Fig. 2C). Additionally, ChIP-qPCR analysis showed the similar ANG-binding pattern as in HeLa cells (Fig. 2D).

### ANG Regulates the Repressive Activity of ABSE

**Figure 3:**
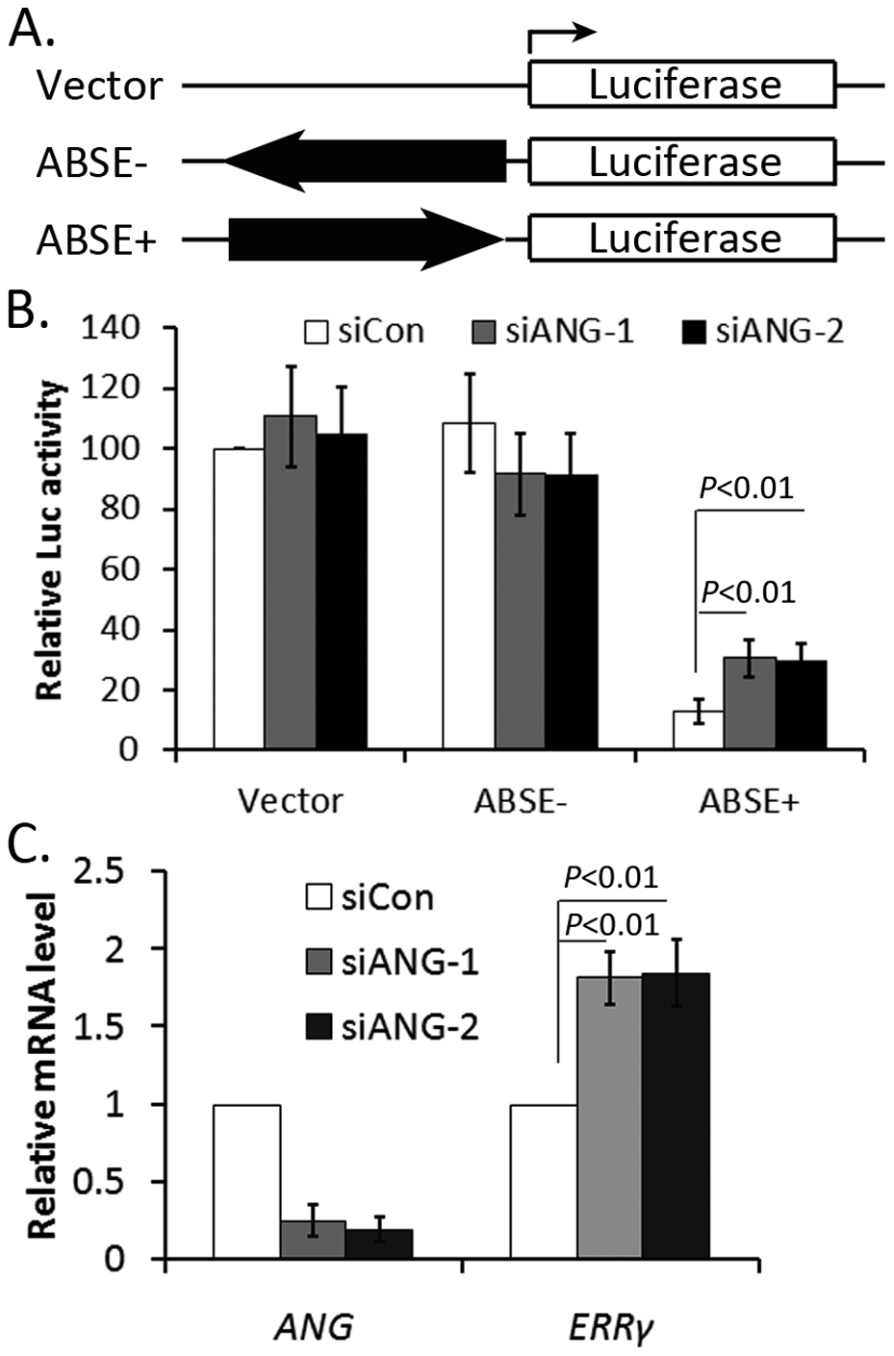
ANG regulates the repressive activity of ABSE and ERRγ expression. (**A**) Schematic illustration of plasmids used in the luciferase assays. (**B**) MCF-7 cells were transfected with the indicated plasmids together with siRNAs targeting control or ANG. Luciferase activities were detected 48 hours after transfection. Relative luciferase activity is a ratio of Firefly luciferse units normalized to Renilla luciferase units. (**C**) MCF-7 cells were treated with siRNA targeting ANG or control for 48 hours. The mRNA levels of *ANG* and *ERRγ* were detected by RT-qPCR.

To test whether this sequence acts as a regulatory element, we cloned it into pGL3-basic vector in the forward (ABSE+) and reverse (ABSE−) direction (Fig. 3A) and detected the role of ANG on its activity in MCF-7 cells. Insertion of ABSE in the reverse direction did not affect luciferase transcription, while its insertion in the forward direction decreased transcription significantly (Fig. 3B), suggesting that ABSE is a transcription repressor. Next, we used the siRNA to silence the endogenous ANG, and the knock down efficiency was identified (Fig. S2). Down-regulation of ANG did not influence the luciferase activities of null vector or ABSE-, but significantly increased the luciferase activity of ABSE+ (Fig. 3B), indicating that the endogenous ANG repressed the activity of ABSE.

### Down-regulation of Endogenous ANG Increased ERRγ Expression

The above data suggested that ANG might repress ERRγ expression at transcription level. We therefore detected the mRNA level of *ERRγ* gene (primers amplifying coding region, Table S1) after down-regulating endogenous ANG expression. Data showed that the mRNA level of *ERRγ* was increased in ANG knocked down cells (Fig. 3C), which confirmed that ANG negatively regulated ERRγ expression and was consistent with the result that ANG repressed the ABSE activity.

### ANG Regulates the Histone Modifications at ABSE Region

**Figure 4:**
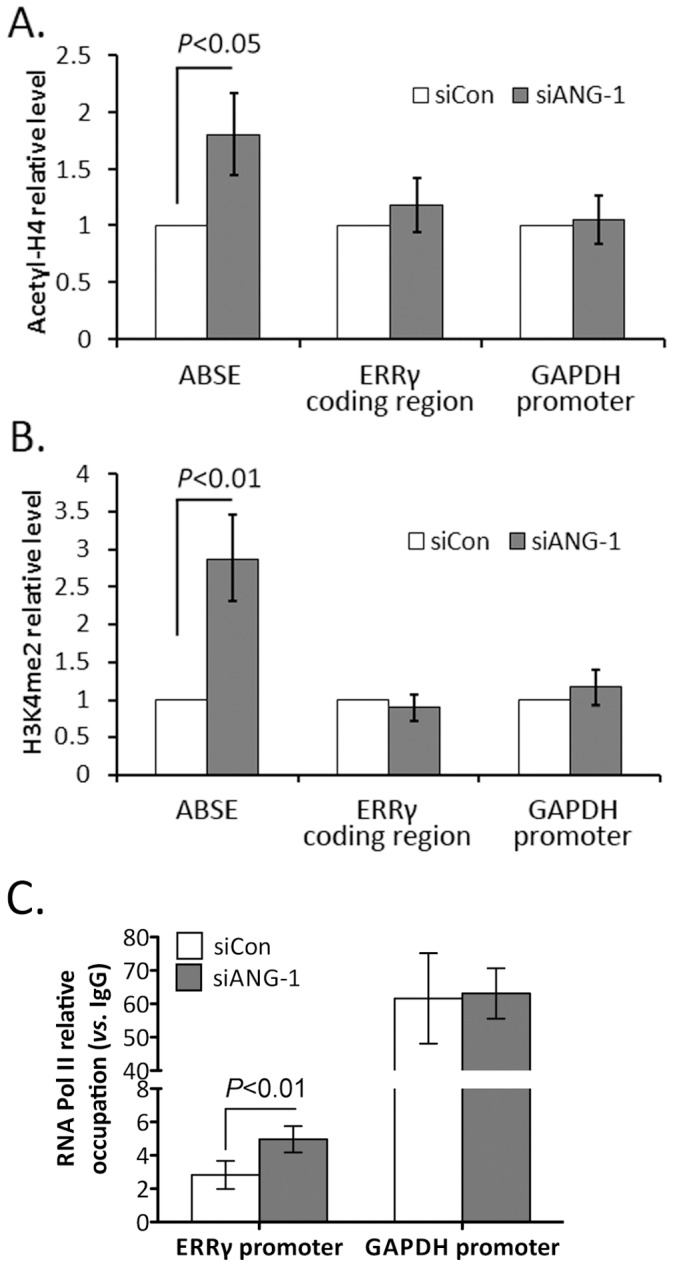
ANG influences the histone modifications and Pol II occupation at ABSE region. MCF-7 cells were transfected with siRNA targeting ANG or control siRNA for 72 hours. ChIP assays were performed with antibodies against acetyl-H4 (**A**), H3K4me2 (**B**), or RNA Pol II (**C**) and analyzed by qPCR. Values were means±s.d. for triplicates.

Epigenetic modifications of histones play a key role in regulating mRNA transcription [17], [18]. To explore whether ANG regulates ERRγ expression through affecting histone modifications at the ABSE region, we employed ChIP assays to analyze the H3K4me2 and acetyl-H4 status. Data showed that knockdown of ANG significantly increased H3K4me2 level and acetyl-H4 level at the ABSE region, but not the ERRγ coding region or GAPDH promoter region (Fig. 4A,B). Since both H3K4me2 and acetyl-H3 are reported as active gene markers [17], [18], our results suggested that ANG may regulate the transcription of ERRγ through an epigenetic mechanism. Consistently, the occupation of RNA Pol II at ABSE region increased when ANG was silenced (Fig. 4C).

### ANG Regulates MCF-7 Cell Proliferation Partially through ERRγ

**Figure 5:**
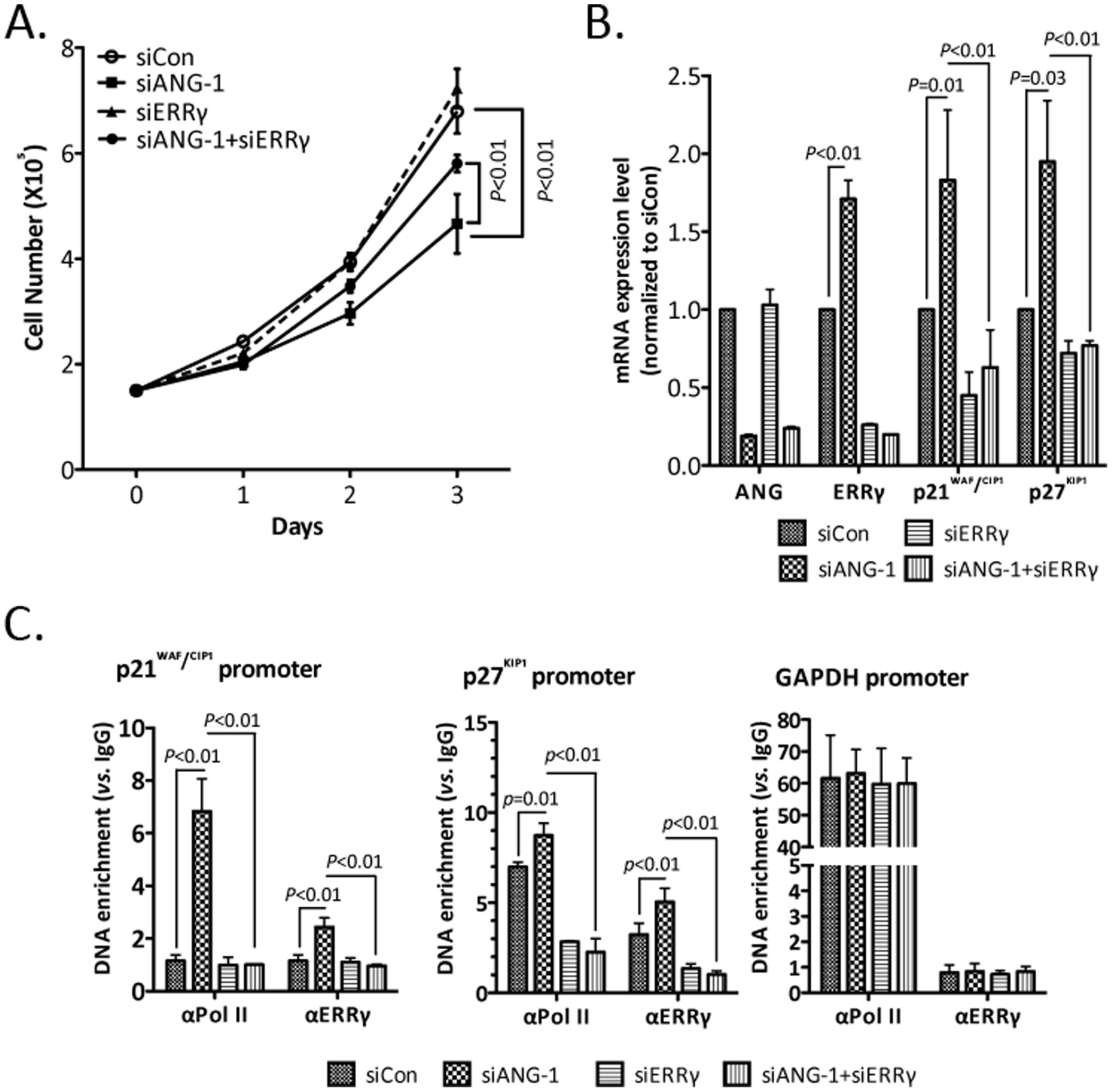
ERRγ is involved in ANG-regulated cancer cell proliferation and cell cycle protein expression. (**A**) MCF-7 cells transfected with siRNAs targeting ANG, ERRγ, or both were seeded at equal density. Cell numbers were counted at each time point as indicated. One-way ANOVA was used for statistical analysis of cell proliferation. (**B**) MCF-7 cells were transfected with siRNAs targeting ANG, ERRγ, or both and the expression levels of indicated genes were detected with RT-qPCR. (**C**) MCF-7 cells transfected with siRNAs targeting ANG, ERRγ, or both were applied to ChIP assays with antibodies against RNA Pol II or ERRγ. The antibody enriched DNA were analyzed by qPCR. Values were means±s.d. for triplicates.

ANG was reported to promote proliferation in many cancer cells. Given our observation that ANG negatively regulates ERRγ expression, we proposed that ERRγ might be involved in ANG-regulated proliferation of breast cancer cells. To test that hypothesis, we down-regulated the expression levels of ANG, ERRγ, or both, and detected the proliferation rate of MCF-7 cells. Knock down of ANG decreased cell proliferation rate. However, the knock down of ERRγ had almost no effect on cell proliferation, which might be because of the low expression level of ERRγ in MCF-7 cells. When both genes were silenced, the proliferation rate of MCF-7 cells increased significantly compared to ANG-deficient cells (Fig. 5A). Prostate cancer cell (PC-3) is another reported target cell line for both ANG [2] and ERRγ [14]. Therefore, we detected the effects of ANG and ERRγ on cell proliferation in this cell line. Data showed that the proliferation inhibition effect of ANG in PC-3 cells can also be rescued by further silencing ERRγ gene (Fig. S3).

### ANG Inhibits the Expressions of ERRγ Target Genes

It has been reported that ERR*γ* suppresses cell proliferation by induction of two cyclin-dependent kinase inhibitors, p21^WAF1/CIP1^ and p27^KIP1^ [14]. We therefore detected the expression levels of these two genes in MCF-7 cells by silencing ANG and ERRγ (Fig. 5B). Data showed that knock down of ANG enhanced the p21^WAF1/CIP1^ and p27^KIP1^ genes expression. The expression levels dropped to the basal level when ERRγ was further silenced. To demonstrate that the expressions of these genes were transcriptionally regulated by ERRγ, we further examined the bindings of ERRγ and Pol II with promoter regions of p21^WAF1/CIP1^ and p27^KIP1^. Knock down of ANG increased the occupations of ERRγ and Pol II on the promoter regions (Fig. 5C). Similar results were gained in PC-3 cells (Fig. S3). These data supported the idea that ERRγ is direct target of ANG and involved in ANG-mediated effects.

### ANG Negatively Correlates with ERRγ in Breast Cancer Tissues

**Figure 6:**
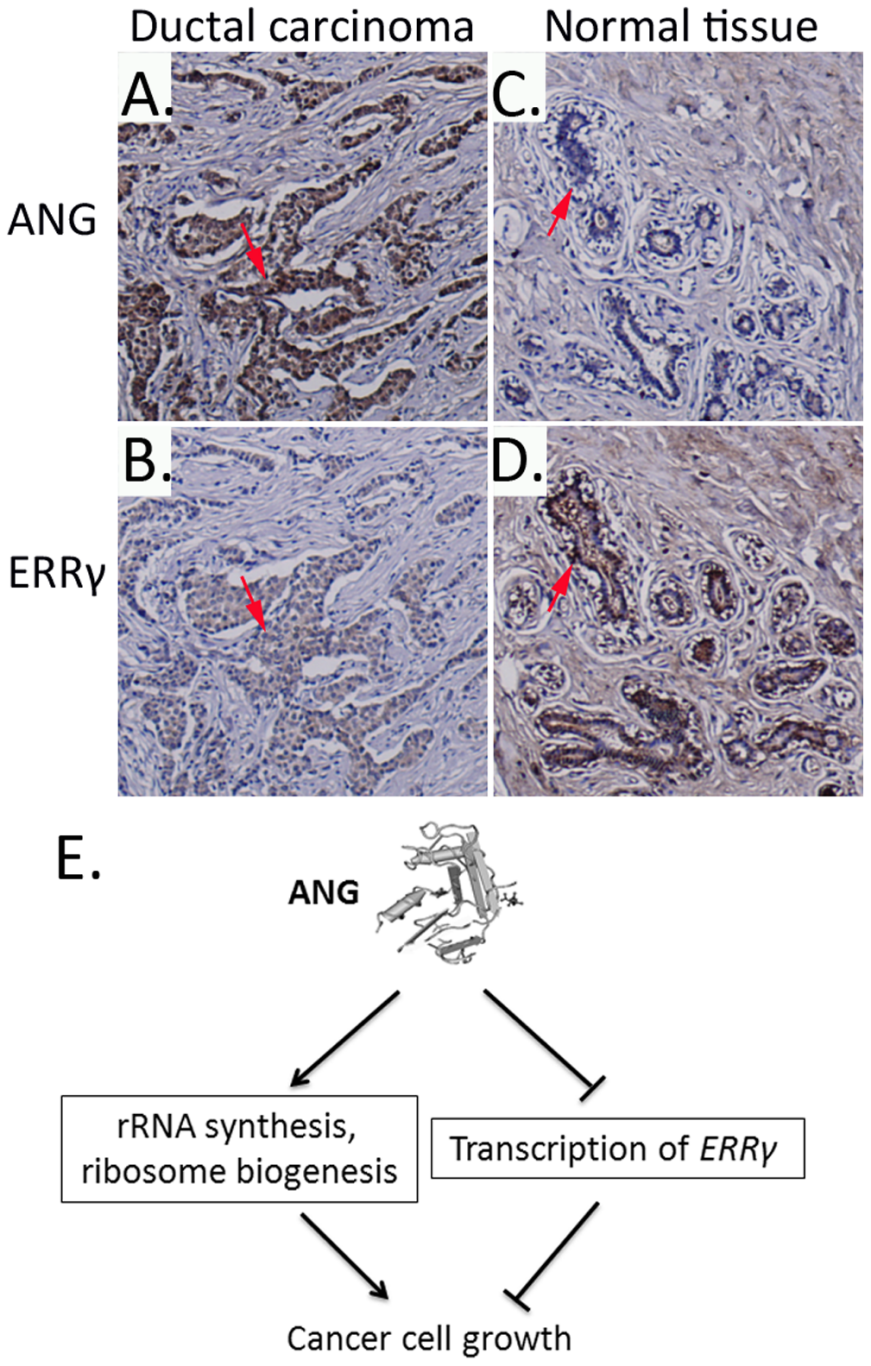
Immunohistochemistry staining of ANG and ERRγ in breast tissue samples. Tissue microarray slide containing human breast ductal carcinoma tissues (**A, B**) and adjacent normal breast tissues (**C, D**) were stained with ANG (**A, C**) or ERRγ (**B, D**) antibody and visualized with Dako’s Envision kit. (Magnification: ×100). (**E**) The roles of ANG in the nucleus. ANG in the nucleolus promotes rRNA production and ribosome biogenesis. Meanwhile, ANG in the nucleoplasm regulates the expressions of its target genes to regulate cancer cell proliferation coordinately.

We next examined the expression levels of ANG and ERRγ by immunohistochemistry (IHC) with a tissue microarray slide containing 4 different human breast ductal carcinoma samples with adjacent normal breast tissues. Representative images are shown in Fig. 6. ANG is almost undetectable in normal ductal epithelial cells but the expression is significantly elevated in breast cancer tissue. On the contrary, the expression level of ERRγ in breast cancer tissue is lower compared to the adjacent normal tissue. We did IHC scoring for each sample (Table S3). The results showed negatively correlation between ANG and ERRγ in paired samples.

## Discussion

Nuclear translocation is essential for ANG-promoted cell proliferation. It has been reported that nuclear ANG is involved in rRNA production [8], [9], and studies also suggest a role of this protein in mRNA transcription regulation [2], [9]. Here, by using a ChIP-cloning method, we identified 12 different ANG-interacting DNA fragments for the first time. Among them, three are located within introns, and nine are upstream or downstream of genes with a distance ranging from 927 bp to 273 kb, further supporting that ANG binds to regulatory elements and is involved in mRNA expression regulation. Future studies on the interactions between ANG with these fragments and the regulatory effects of ANG on the expressions of nearby genes will improve our understanding of other possible biological roles of this growth factor.

We have reported previously that ANG binds to the CT repeats in rDNA region [9]. We searched the 12 ANG-binding elements and did not find any typical CT repeats, implying that nuclear angiogenin may bind to other DNA sequences. This is conceivable because one target cell can take up to 1.3×10^6^ ANG molecules and binding to the CT repeat would not account for all of the ANG molecules present in the nucleus [19]. We next asked whether there are any conserved sequences among the 12 fragments revealed by ChIP. Alignment of these fragments showed no consensus elements (data not shown). There are two possible explanations for the absence of a specific manner in ANG/DNA binding. First, ANG may serve as a structural protein and regulate transcription by influencing DNA availability to polymerases, just like high mobility AT-hook 2 (HMGA2) [20] and upstream binding factor (UBF) [21]. Second, ANG might bind to chromatin through additional factors since ANG can interact with many transcription factors in the nucleus, such as FUBP1, UHRF1, YBX1, SUB1, as shown in our previous co-imuunoprecipitation mass spectrometry data [22]. We also analyzed the transcription factor consensus binding sites on the ABSE by PROMO online program [23] (Fig. S4). A series of binding elements of transcription factors, such as NF-1, SP-1 GATA-1, GR, CREB, were predicted on ABSE. These results suggested that ABSE was a regulatory sequence, and ANG may interact with these transcription factors to regulate the ABSE activity. However, we still do not know how ANG specifically recognizes its target genes. This question can be solved when more ANG-binding sequences are identified and mutation studies in these sequences are carried out in the future.

Interestingly, two of the fragments contained the same sequence upstream of ERRγ gene. Our data showed that the sequence is a repressive element. ANG regulates its activity and ERRγ expression in breast cancer cells. ERRγ has been reported to inhibit the development of prostate cancer [14] and breast cancer [15] in mouse models. It is shown that ERRγ inhibited cancer cell proliferation by increasing expression of two cyclin-dependent kinase inhibitors p21^WAF/CIP^ and p27^KIP1^ [14]. Our data showed that ANG-promoted cell proliferation and inhibited the expression levels of p21^WAF/CIP^ and p27^KIP1^ through ERRγ, as knockdown of ERRγ can increase the proliferation rate in ANG-deficient cells. Therefore, the nucleolar and nucleoplasmic ANG may work coordinately to regulate cancer cell proliferation. Nucleolar ANG increase rRNA production and ribosome biogenesis to meet the high demand of protein synthesis. Meanwhile, nucleoplasmic ANG inhibits ERRγ expression and then increases p21^WAF/CIP^ and p27^KIP1^ expression, thereby promotes cell cycle transition (Fig. 6E).

It should be noted that the inhibitory effect of ANG on ERRγ expression is cell type dependent, as neither the expression level of ERRγ nor the histone modifications at ABSE region changed after ANG knockdown in HeLa cells (data not shown), although ANG can bind to ABSE in this cell line. ABSE also showed strong repressive activity in HeLa cells but its activity did not change when ANG was knocked down (Fig. S5). All these data suggested that ANG might need other factors to regulate ERRγ transcription. The different effects of ANG in MCF-7 and HeLa cells might be caused by the availability of the regulatory factors.

How does ANG regulate ERRγ expression? Our data showed that knockdown of ANG increased active gene markers H3K4me2 and acetyl-H4 at ABSE region, therefore the presence of ANG may induce heterochromatin formation, fasten the rDNA structure and consequently exclude the recruitment of transcriptional machineries. Since ANG is not known to have histone modifying activities, it may induce epigenetic changes through recruiting other modifying enzymes including histone methylation and de-acetylation enzymes. Further studies on the relationships between ANG and histone-modifying enzymes will facilitate to elucidate the detailed mechanism of how ANG regulates ERRγ transcription.

## Supporting Information

Figure S1 **Sonication of chromatin DNA to 600 base pairs.** HeLa cell chromatin DNA was sonicated, DNA was then extracted and separated by agarose gel.
(TIF)

Figure S2 **Knockdown efficiency of ANG.** HeLa cells were transfected with control siRNA or siRNA targeting ANG. Cells were harvested 48 hours after transfection and ANG protein levels were detected by immunoblotting.
(TIF)

Figure S3 **ERRγ is involved in ANG-regulated cancer cell proliferation and cell cycle protein expression in PC-3 cells.** (**A**) PC-3 cells transfected with siRNAs targeting ANG, ERRγ, or both were seeded at equal density. Cell numbers were counted at each time point as indicated. One-way ANOVA was used for statistical analysis of cell proliferation. (**B**) PC-3 cells were transfected with siRNAs targeting ANG, ERRγ, or both and the expression levels of indicated genes were detected with RT-qPCR. (**C**) PC-3 cells transfected with siRNAs targeting ANG, ERRγ, or both were applied to ChIP assays with antibodies against RNA Pol II or ERRγ. The antibody enriched DNA were analyzed by qPCR. Values were means±s.d. for triplicates.
(TIF)

Figure S4 **Transcription factor consensus binding sites on the ABSE.** The transcription factor consensus binding sites on the ABSE was analyzed by PROMO online program.
(TIF)

Figure S5 **Knockdown of ANG in HeLa cells does not change ABSE activity.** HeLa cells were transfected with pGL3-basic or pGL3-ABSE together with siRNAs targeting control or ANG. Luciferase activities were detected 48 hours after transfection. Relative luciferase activity is a ratio of Firefly luciferse units normalized to Renilla luciferase units.
(TIF)

Table S1 **Information of primers used in this study.**
(XLS)

Table S2 **Sequence of ANG-binding fragments identified by ChIP-cloning.**
(XLS)

Table S3 **IHC scoring of ANG and ERRγ expressions in 4 breast ductal carcinoma samples.**
(DOC)
