# Supplementary figures and images for: Identification of Estrogen Receptor-Related Receptor Gamma as a Direct Transcriptional Target of Angiogenin
Source: PLoS One. 2013 Aug 15;8(8):e71487. doi: 10.1371/journal.pone.0071487 (PMC3744552; doi:10.1371/journal.pone.0071487)

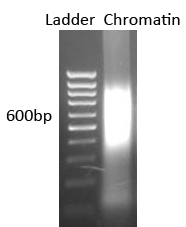

Supplement: Figure S1 — Sonication of chromatin DNA to 600 base pairs. HeLa cell chromatin DNA was sonicated, DNA was then extracted and separated by agarose gel. (TIF) [file pone.0071487.s001.tif]

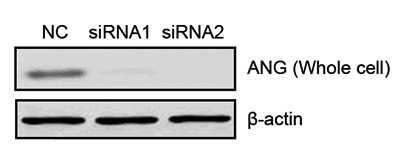

Supplement: Figure S2 — Knockdown efficiency of ANG. HeLa cells were transfected with control siRNA or siRNA targeting ANG. Cells were harvested 48 hours after transfection and ANG protein levels were detected by immunoblotting. (TIF) [file pone.0071487.s002.tif]

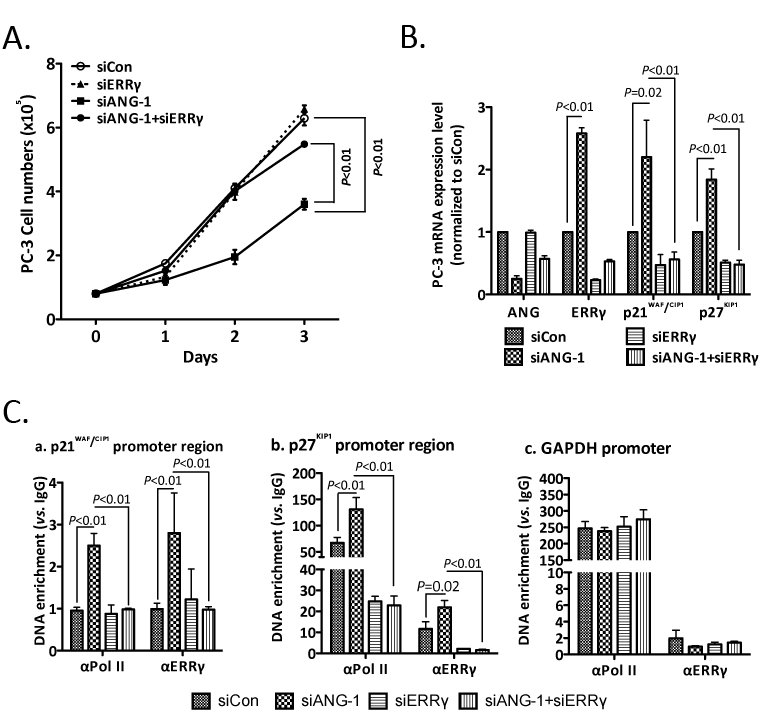

Supplement: Figure S3 — ERRγ is involved in ANG-regulated cancer cell proliferation and cell cycle protein expression in PC-3 cells. (A) PC-3 cells transfected with siRNAs targeting ANG, ERRγ, or both were seeded at equal density. Cell numbers were counted at each time point as indicated. One-way ANOVA was used for statistical analysis of cell proliferation. (B) PC-3 cells were transfected with siRNAs targeting ANG, ERRγ, or both and the expression levels of indicated genes were detected with RT-qPCR. (C) PC-3 cells transfected with siRNAs targeting ANG, ERRγ, or both were applied to ChIP assays with antibodies against RNA Pol II or ERRγ. The antibody enriched DNA were analyzed by qPCR. Values were means±s.d. for triplicates. (TIF) [file pone.0071487.s003.tif]

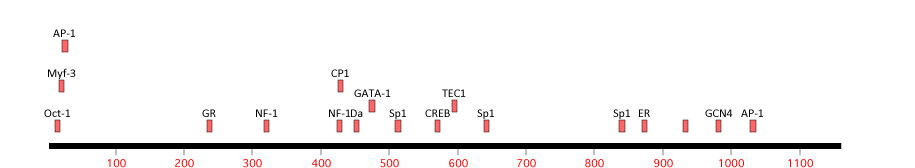

Supplement: Figure S4 — Transcription factor consensus binding sites on the ABSE. The transcription factor consensus binding sites on the ABSE was analyzed by PROMO online program. (TIF) [file pone.0071487.s004.tif]

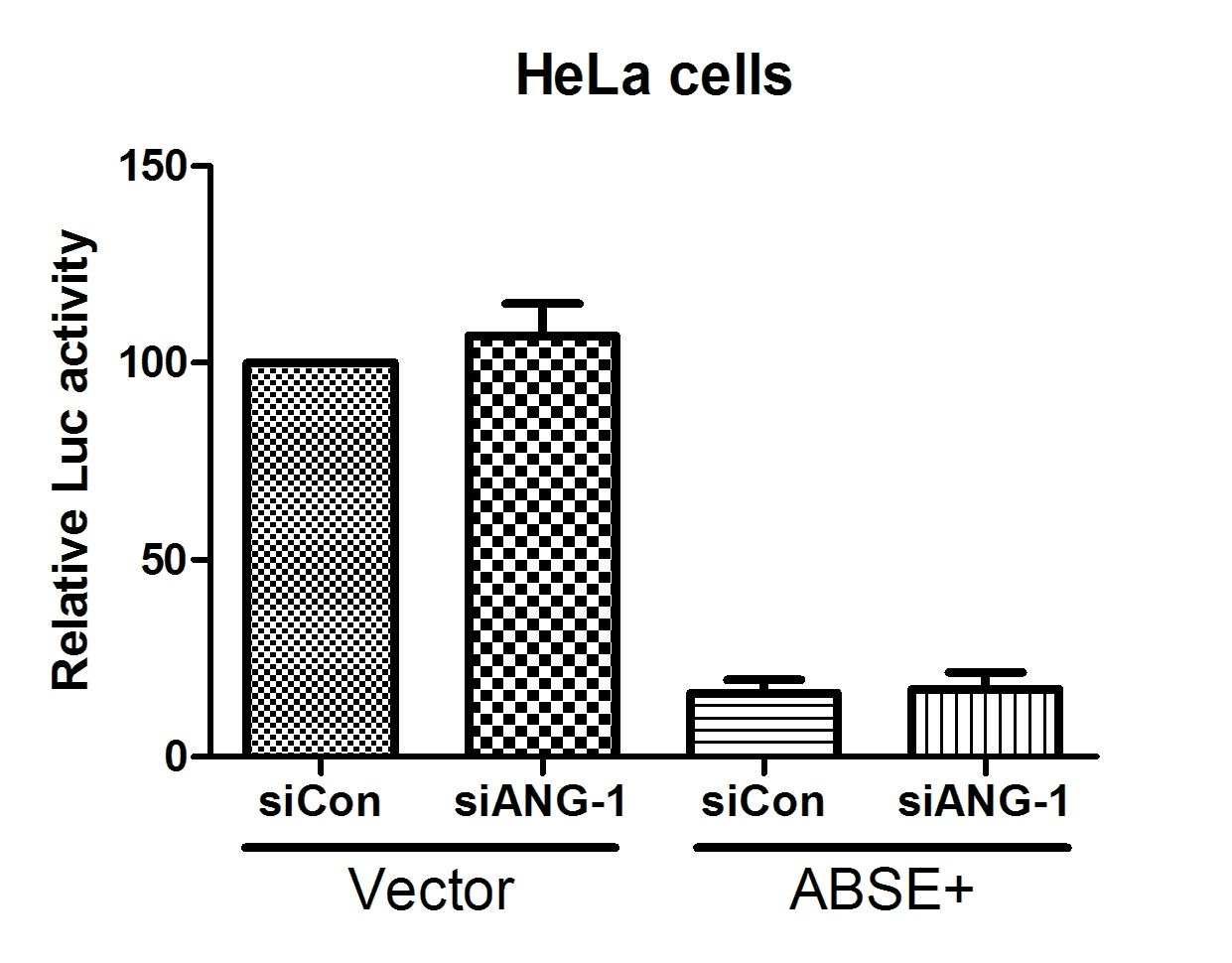

Supplement: Figure S5 — Knockdown of ANG in HeLa cells does not change ABSE activity. HeLa cells were transfected with pGL3-basic or pGL3-ABSE together with siRNAs targeting control or ANG. Luciferase activities were detected 48 hours after transfection. Relative luciferase activity is a ratio of Firefly luciferse units normalized to Renilla luciferase units. (TIF) [file pone.0071487.s005.tif]
