# Supplementary material for: Identification of Estrogen Receptor-Related Receptor Gamma as a Direct Transcriptional Target of Angiogenin
Source: PLoS One. 2013 Aug 15;8(8):e71487. doi: 10.1371/journal.pone.0071487 (PMC3744552; doi:10.1371/journal.pone.0071487)
Supplement: Table S3 — IHC scoring of ANG and ERRγ expressions in 4 breast ductal carcinoma samples. (DOC) [file pone.0071487.s008.doc]

**Table S3. IHC indexing of breast cancer samples.**

|  | Patient 1 | | Patient 2 | | Patient 3 | | Patient 4 | |
| --- | --- | --- | --- | --- | --- | --- | --- | --- |
|  | Cancer | Normal | Cancer | Normal | Cancer | Normal | Cancer | Normal |
| ANG | ++++ | + | +++ | + | +++ | - | ++ | - |
| ERRγ | + | +++ | + | ++ | + | ++ | ++ | +++ |

- not detectable; + low; ++ moderate; +++ high; ++++ very high
